# Design of a PDZbody, a bivalent binder of the E6 protein from human papillomavirus

**DOI:** 10.1038/srep09382

**Published:** 2015-03-23

**Authors:** O. Andreas Karlsson, Juan Ramirez, Daniel Öberg, Tony Malmqvist, Åke Engström, Maria Friberg, Celestine N. Chi, Mikael Widersten, Gilles Travé, Mikael T. I. Nilsson, Per Jemth

**Affiliations:** 1 Department of Medical Biochemistry and Microbiology, Uppsala University, BMC Box 582, SE-75123 Uppsala, Sweden; 2 Biotechnologie et Signalisation Cellulaire UMR 7242, Ecole Supérieure de Biotechnologie de Strasbourg, Boulevard Sébastien Brant, BP 10413, F-67412 Illkirch, France; 3 Department of Chemistry-BMC, Uppsala University, Box 576, SE-751 23 Uppsala, Sweden

## Abstract

Chronic infection by high risk human papillomavirus (HPV) strains may lead to cancer. Expression of the two viral oncoproteins E6 and E7 is largely responsible for immortalization of infected cells. The HPV E6 is a small (approximately 150 residues) two domain protein that interacts with a number of cellular proteins including the ubiquitin ligase E6-associated protein (E6AP) and several PDZ-domain containing proteins. Our aim was to design a high-affinity binder for HPV E6 by linking two of its cellular targets. First, we improved the affinity of the second PDZ domain from SAP97 for the C-terminus of HPV E6 from the high-risk strain HPV18 using phage display. Second, we added a helix from E6AP to the N-terminus of the optimized PDZ variant, creating a chimeric bivalent binder, denoted PDZbody. Full-length HPV E6 proteins are difficult to express and purify. Nevertheless, we could measure the affinity of the PDZbody for E6 from another high-risk strain, HPV16 (*K*_d_ = 65 nM). Finally, the PDZbody was used to co-immunoprecipitate E6 protein from HPV18-immortalized HeLa cells, confirming the interaction between PDZbody and HPV18 E6 in a cellular context.

At present, around 200 different types of human papillomaviruses (HPVs) have been documented^1^, and a subset of these may cause cancer if the infection is not cleared from the body^2^. During infections the virus hijacks the cell by expressing certain viral proteins, including HPV E6 and E7, which among other things promote degradation of p53, Retinoblastoma tumour suppressor protein and PDZ-domain containing proteins, respectively. Continued expression of HPV E6 and E7 could lead to transformation of the infected cell^3^. Importantly, the transformed cell is only viable as long as the two proteins HPV E6 and E7 are expressed^4^ and the HPV E6 protein is therefore considered a promising drug target^5^. For example, if expression of HPV E6 is suppressed, levels of p53 will rise and the HPV-immortalized cancer cell may go into apoptosis. Repression of HPV E6^6^,^7^,^8^, including experiments using sonoporation of antibodies against HPV E6^9^ as well as siRNA silencing of HPV E6 mRNA^10^ have demonstrated the feasibility of the approach.

While antibodies work well in many cases, there is a general interest in exploring alternative scaffolds for designing protein binders^11^,^12^. Inspired by previous work on bivalent inhibitors^13^,^14^,^15^ including studies in our lab^16^,^17^,^18^ we have here developed a high-affinity chimeric protein binder of HPV E6 in two steps: (*i*) by optimizing the affinity of one natural E6 ligand using phage display, namely PDZ2 from Synapse associated protein 97 (SAP97) and (*ii*) linking an alpha helix from another natural ligand of the E6 protein, the ubiquitin ligase E6-associated protein (E6AP)^19^,^20^,^21^ to the optimized PDZ domain. This strategy of linking two binding epitopes that have two distinct binding sites in their common target will usually result in an increased affinity for the target. The binding of the first epitope is an *intermolecular* interaction with an affinity similar to that of the isolated epitope, but the second binding event will be an *intramolecular* interaction. Herein lies the strength of multivalent interactions: the high “efficient concentration” of the second epitope may substantially increase the affinity of the linked, bivalent molecule for the target, as compared to the affinities of the individual epitopes^15^. However, how much the affinity is improved by linking two epitopes is hard to predict and depends on several factors such as linker length, interactions between linker and the protein target, and conformational constrains. The first part of the design, to find two binding epitopes, which can be linked, may also prove difficult. We have employed the attractive and straightforward strategy of using natural cellular interaction partners of our target, the HPV E6 protein. Indeed, the resulting bivalent binder (denoted PDZbody) has an affinity towards HPV16 E6 of around 65 nM and it can be used in co-immunoprecipitation experiments to detect HPV18 E6 in HeLa cells.

## Results and Discussion

Design of inhibitors for protein-protein interactions is a rapidly developing field^22^. Whereas potent small molecule inhibitors for enzymes are relatively easy to design they are less efficient in protein-protein interactions, although a recent study shows encouraging results for HPV16 E6^23^. Nevertheless, peptidomimetics and protein drugs are promising as drug candidates for multipartner-binding proteins like the oncogenic HPV E6, given the high possibility of specificity in protein-protein interactions. To this end we have designed a bivalent protein binder of HPV E6, a chimera between a PDZ domain and a helix from E6AP that we call PDZbody (“PDZ-based antibody”).

### Improving the affinity of a PDZ domain for HPV18 E6 by phage display

The C-termini of high risk HPV E6 proteins interact with PDZ domains from different proteins^24^, for example SAP97 (also called human Dlg)^25^,^26^. X-ray and NMR studies show that the C-terminus of high-risk HPV E6 proteins binds to the peptide binding groove of the PDZ domain in a so-called canonical fashion, i.e., as a β-strand to form an extended anti-parallel β-sheet with the PDZ domain^27^,^28^. We have previously characterized the interaction between the C-terminal domain of E6 proteins, or peptides corresponding to C-termini, and different PDZ domains^29^,^30^,^31^. The inherent affinity between SAP97 PDZ2 and the C-terminus of HPV18 E6 was particularly high (0.4 μM)^30^. This PDZ domain has been thoroughly investigated with regard to both binding^29^,^31^ and folding^32^,^33^ and a crystal structure of the pseudo wild type SAP97 PDZ2 used in our studies is available (protein data bank code 2X7Z)^32^. It was therefore chosen as an appropriate protein scaffold for the design of an HPV E6 binder. The pseudo wild type SAP97 PDZ2 contains two mutations: C378A to avoid formation of disulphide bridges and I342W as a probe for fluorescence and absorbance^29^. PDZ domains usually display affinities in the range 1-100 μM for natural ligands^34^,^35^. However, we reasoned that we could further improve the affinity between this PDZ domain and the HPV18 E6 C-terminus, based on previous phage display experiments on other PDZ domains^36^,^37^.

**Figure 1:**
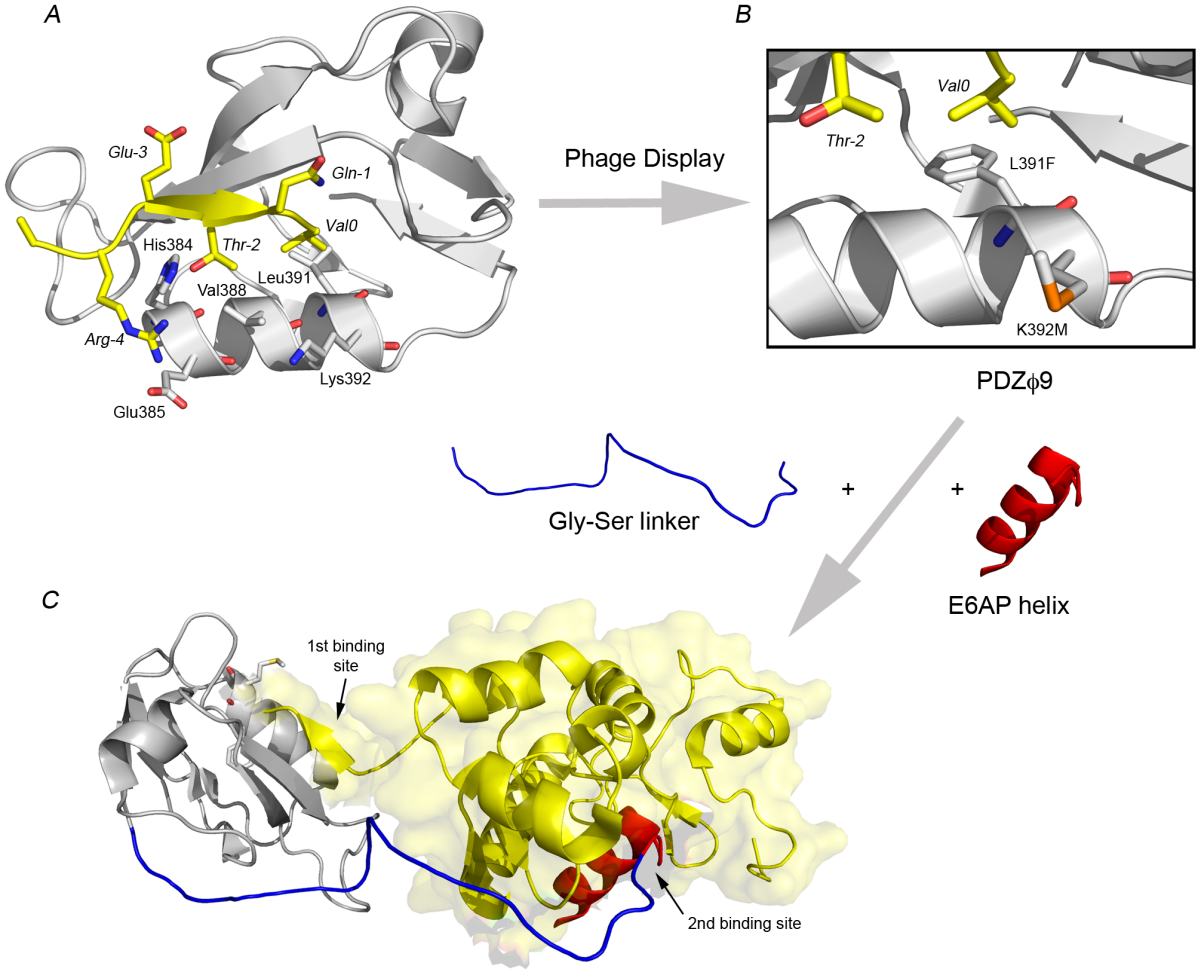
Strategy for designing a high affinity binder of HPV E6. (*A*) Crystal structure of SAP97 PDZ2 with bound peptide (RRRETQV) corresponding to the C-terminus of HPV18 E6 (Protein data bank code: 2I0L). The five highlighted positions in the α-helix were included in the phage library. PDZ domains usually bind the C-terminus of target proteins and the last residue, in this case a valine (*Val0*) is important for the affinity and specificity of the interaction. The numbering of the peptide is according to convention in the PDZ field and peptide residues -4 to 0 correspond to residues 154–158, respectively, in HPV18 E6. (*B*) The best binder obtained after the phage display selection (PDZϕ9) contained two substitutions as compared to the pseudo wild type SAP97 PDZ2, namely L391F and K392M. Leu391 is part of the hydrophobic pocket, which binds the side-chain of the C-terminal residue. It is likely that a Phe in this position results in a better fit of *Val0* in the pocket. (*C*) To increase the affinity for HPV E6 (yellow) further, the E6AP helix (red) was attached to the N-terminus of PDZϕ9 (grey) via a Gly-Ser linker (blue). The E6AP helix binds in between the two domains of HPV E6 and provides a second interaction site for the resulting PDZbody. This hypothetical model of the complex between PDZbody20 and HPV E6 is based on the crystal structures of HPV16 E6 with the E6AP helix (protein data bank code: 4GIZ) and that of SAP97 PDZ2.

A phage display library of the PDZ domain was thus designed as follows. Five positions in the peptide binding pocket (His384, Glu385, Val388, Leu391, Lys392) were selected based on their interactions with the peptide in the crystal structure between SAP97 PDZ2 and RRRETQV (Protein data bank code: 2I0L, Fig. 1)^27^. A DNA library coding for the PDZ domain was designed such that each of these five positions could encode all amino acid residues except Cys (to avoid disulphide bridges), resulting in a theoretical library size of 2.47×10^6^ unique members. The DNA library was ligated into a modified version of the pComb3 phagemid^38^ such that the expressed PDZ domain was C-terminally fused via a short linker to the truncated geneIII protein. This arrangement allows each phage to present on average one PDZ domain library member monovalently to the surrounding environment. The final phage library was constructed by transformation of the plasmids into *Escherichia coli* XL1-Blue cells. The number of transformants was roughly ten times that of the unique members, allowing for complete coverage of all possible variants in the selection.

Successful panning for a novel PDZ domain with increased affinity for HPV18 E6 protein was carried out with the C-terminal domain of HPV18 E6 (residues 82-158) linked via the N-terminus to His-tagged lipoyl domain^39^ (Lipo-E6_18_-C). This construct was immobilized via the His-tag on paramagnetic precharged nickel particles. We started with roughly 10^12^ phages (ϕ_in_) in the first round of selection and the enrichment of phages after each round was monitored as the ratio of number of survivors over the number of phages entering the selection, (ϕ_out_/ϕ_in_). The ratio ϕ_out_/ϕ_in_ started to grow after the second round and levelled off after round 5 (5.9×10^−5^, 1.1×10^−5^, 6.5×10^−5^, 1.7×10^−4^ and 2.9×10^−4^ for rounds one to five, respectively). After round 5 the panning was stopped and the PDZ domain sequences were analysed for 24 survivors. Among the 24 sequenced variants, 9 distinct sequences were present, as well as the wild type (Table 1).

**Figure 2:**
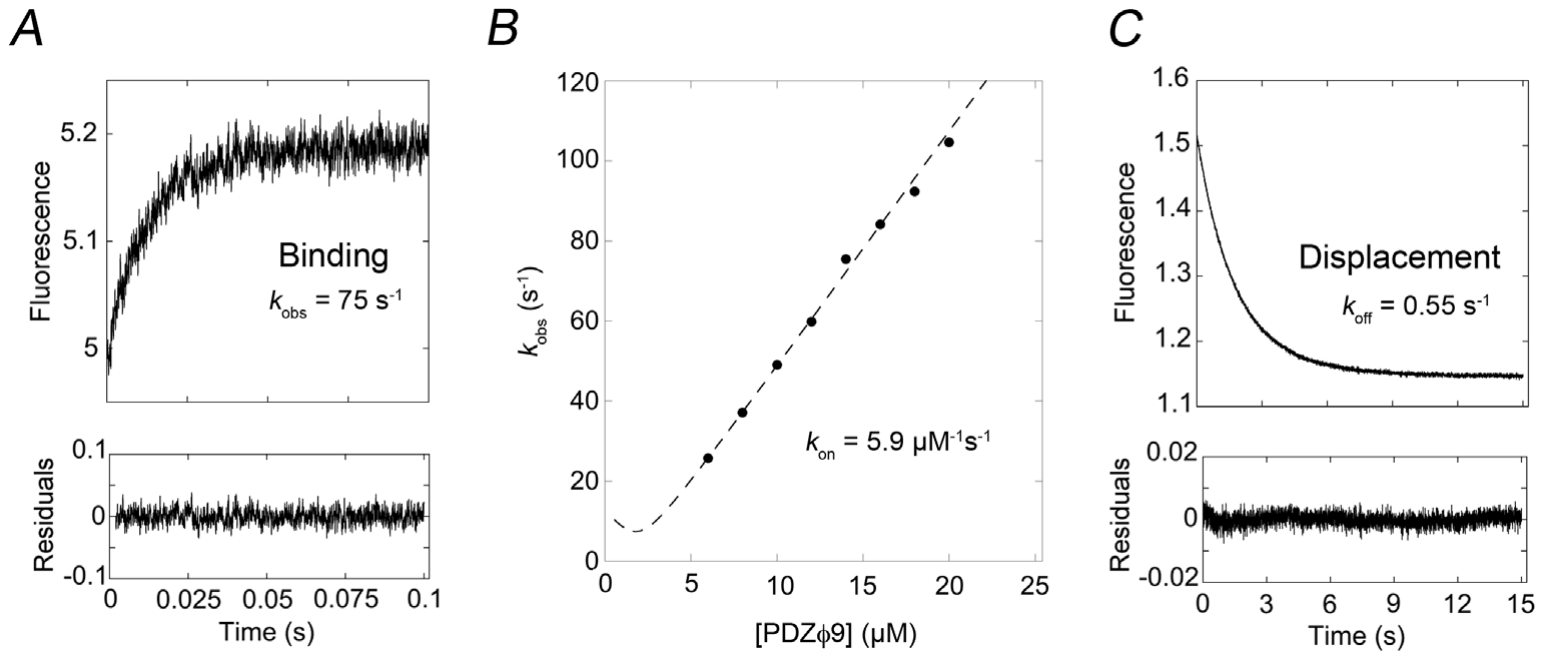
Binding data for PDZϕ9 determined by stopped-flow spectroscopy. (*A*) The experimental binding trace was obtained by mixing 14 μM PDZϕ9 variant with 2 μM of Lipo-E6_18_-C. The trace was fitted to a single exponential function to obtain the observed rate constant *k*_obs_. (*B*) Observed rate constants were plotted *versus* different concentrations of PDZϕ9 to obtain the association rate constant *k*_on_ as the slope of the curve. (*C*) The dissociation rate constant *k*_off_ was determined separately in a displacement reaction, in which a pre-formed complex between the PDZϕ9 (0.75 μM) and the C-terminal domain of HPV18 E6 (0.75 μM) was mixed with a large excess of dansyl-labeled peptide corresponding to the C-terminal six residues of HPV18 E6 (300 μM). The peptide competes for binding to the PDZ domain and gives a distinct signal on binding. At high concentration of peptide, none of the dissociated C-terminal domain of HPV18 E6 will re-bind, and the *k*_obs_ value of the trace is equal to the overall dissociation rate constant, *k*_off_. The *K*_d_ value is then calculated by taking *k*_off_/*k*_on_. See Table 1 for rate constants and *K*_d_ values for PDZ variants.

The survivors of the phage display selection were expressed, purified and subjected to binding studies using stopped-flow spectroscopy to measure the affinity for the C-terminal domain of HPV18 E6 (Table 1, Fig. 2). The affinity of the best binder (PDZϕ9) after phage display selection (93 nM) is good considering the affinities generally observed (>1 μM) for supposedly natural PDZ-peptide interactions^35^,^40^ or designed ones^36^,^37^,^41^. The increase in affinity was due to a decrease in the dissociation rate constant *k*_off_, suggesting a better complementarity in the binding interface. The three best PDZ variants all contained the L391F mutation, which results in a larger side-chain in the hydrophobic pocket of the PDZ domain. Possibly, this leads to more favourable hydrophobic interactions with the last residue of the HPV E6. The three best variants also contained mutation of Lys392 into a non-charged residue. The effect of this mutation is less clear but could involve fewer unfavourable long-range interactions with the Arg side-chains of HPV18 E6 at positions 152, 153 and 154, which are situated adjacent to the four residues in the ligand binding pocket of the PDZ domain (see Figure 1A).

### Creating a bivalent high affinity binder towards HPV E6

Linking two binding epitopes is an efficient strategy to increase affinity towards a target. Hereby the effective concentration is increased for the second part of the inhibitor once the first one is bound^15^. The cancer-causing high-risk HPV E6 proteins are perfect targets for bivalent inhibitors. The disordered C-terminus of HPV E6 proteins binds to a peptide-binding groove on the PDZ domain to form an extended β-sheet^27^. The HPV E6 protein has another well defined interaction site for α-helices carrying the LxxLL motif^19^, which binds between the two domains of the HPV E6 protein^42^ with μM affinity^43^,^44^. By first optimizing the PDZ peptide-binding groove with phage display and then combining this optimized PDZ domain (PDZϕ9) with an LxxLL helix from E6AP we created chimeric constructs denoted PDZbodies (Fig. 1).

Based on the crystal structure^42^, we designed PDZbodies in which the LxxLL-motif containing helix from E6AP was attached to the N-terminus of PDZϕ9 via a Gly-Ser linker. It was difficult to estimate the optimal length of the linker because (*i*) the C-terminus of HPV E6 is disordered and (*ii*) the effect of direct interactions between the linker and HPV E6 is impossible to predict. Four different constructs were therefore engineered with total linker lengths of 8, 15, 20 and 25 residues, respectively, counting from the E6AP helix to the first β-strand of the PDZ domain, as defined in the structure of the pseudo wild type SAP97 PDZ2, solved without bound peptide^32^.

**Figure 3:**
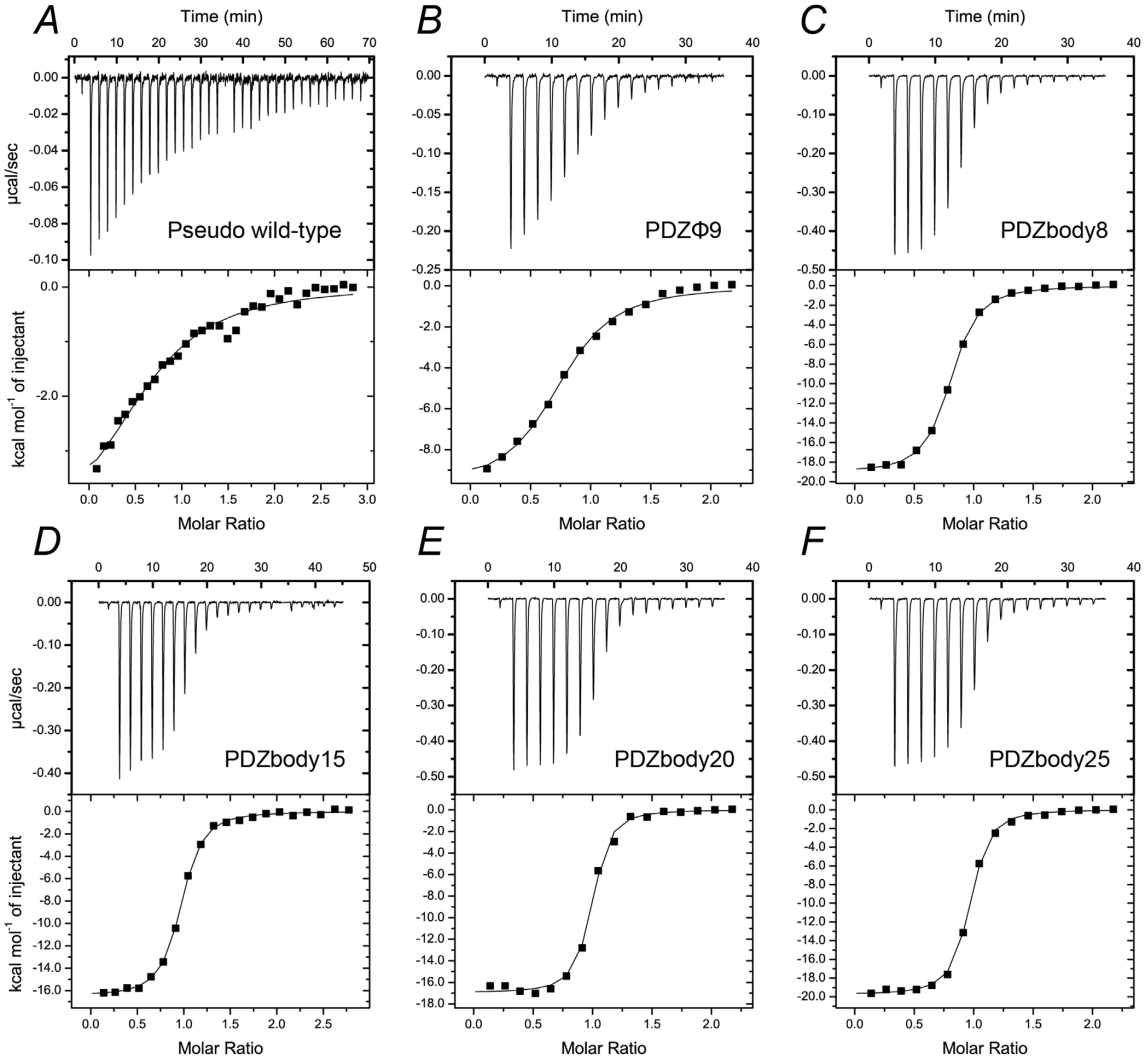
Effect of adding the E6AP helix to the N-terminus of the best binder after phage display, PDZϕ9. ITC experiments of binding between full-length HPV16 E6 protein and (*A*) pseudo wild type SAP97 PDZ2, (*B*) PDZϕ9, (*C*) PDZbody8, (*D*) PDZbody15, (*E*) PDZbody20 and (*F*) PDZbody25. See Table 2 for fitted parameters for all variants.

To evaluate the effect of the helix on the affinity, binding of full-length HPV E6 to the four PDZbodies as well as to PDZϕ9 and the original pseudo wild type SAP97 PDZ2 was measured by isothermal titration calorimetry (ITC) (Fig. 3, Table 2). HPV E6 proteins are very difficult to express in a soluble monomeric form and HPV18 E6 is particularly problematic in this respect^42^. We therefore resorted to use full-length HPV16 E6 for these binding experiments^45^,^46^. The difference in sequence between HPV16 and HPV18 E6 that gives the main difference in affinity and specificity for PDZ domains is the C-terminal residue, which is Leu in HPV16 E6 and Val in HPV18 E6^47^. First, we note that the affinity of PDZϕ9 towards HPV16 E6 (Table 2) was increased by the same factor as for the HPV18 E6 C-terminal domain (5-6-fold, Table 1). Secondly, and what is important for the design, there was a clear positive effect on the *K*_d_ value upon addition of the E6AP helix to PDZϕ9. The best effect was seen for PDZbodies with the longer linkers 15, 20 and 25, with PDZbody20 displaying the lowest *K*_d_ value (65 nM) and thus a 14-fold improved affinity (Table 2). The increase in affinity, resulting from an additional 1–1.6 kcal/mol in favourable free energy, was due to an increase in the enthalpy of binding Δ*H*, consistent with extra binding energy originating from the new interaction surface between the helix and the HPV16 E6 protein. The observed binding stoichiometry was between 0.75–0.94, which is in good agreement with the expected 1:1 ratio between PDZbody/PDZ domain variant and HPV16 E6, respectively. HPV16 and 18 are both high-risk strains with a similar overall structure of their respective E6 protein^42^,^47^. Whilst the affinity of PDZϕ9 for the C-terminus of HPV18 E6 is around 10 times higher than that for HPV16 E6, we can only speculate about the effect of the linked helix on the affinity between PDZbody20 and full-length HPV18 E6. Nevertheless, one of the highest natural affinities measured for PDZ domains is that between wild-type SAP97 PDZ2 and HPV18 E6 C-terminal domain (0.4 μM)^30^, and the measured affinity for HPV16 E6 and PDZbody20 is 6-fold higher than this.

### Using the PDZbody to detect HPV18 E6 from HeLa cells

**Figure 4:**
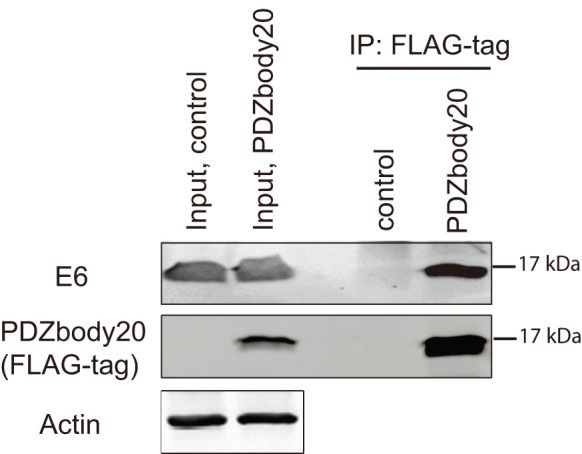
HPV18 E6 is pulled down by PDZbody20. HeLa-cells were transfected with either a control CMV vector expressing the triple FLAG-tag or the same vector expressing the pPDZbody20 with a triple FLAG-tag. After 24 h the cells were harvested and the lysate was incubated with agarose beads with anti-FLAG-tag antibodies to immunoprecipitate (IP) the PDZbody20. After elution, samples were run on denaturing polyacrylamide gels, followed by western blotting using antibodies to HPV18 E6 and FLAG-tag. Actin was used as a control to ensure equal protein amounts in the input lysate. Two separate gels were run and each blotted independently for either HPV18 E6 or FLAG-tag. Input levels represent 5% of total protein lysate used for co-immunoprecipitation.

HeLa cells are HPV18-immortalized cells and consequently express the HPV18 E6 protein to suppress p53^48^. By transfection we overexpressed a construct with the PDZbody20 fused to a triple FLAG-tag in HeLa cells. After 24 h the cells were lysed and the FLAG-tagged PDZbody20 was captured by agarose beads carrying an antibody for the FLAG-tag. The beads were washed and bound proteins released by boiling and separated by SDS-PAGE. The proteins were subsequently transferred to a membrane by western blotting and detected using antibodies against HPV18 E6, the FLAG-tag and actin, respectively. Fig. 4 clearly shows that the PDZbody20 is able to co-immunoprecipitate the HPV18 E6 protein in HeLa cells. The PDZbody20 migrated near the 17 kDa protein marker on SDS-PAGE gels which corresponds well with the theoretical molecular weight of 16.6 kDa (158 amino acids) for this protein. These results indicate that the PDZbody 20 can be used to efficiently detect HPV18 E6 and suggest an interaction between the two proteins in HeLa cells.

### Concluding remarks

HPV E6 proteins promote degradation of p53 by recruiting the ubiquitin ligase E6AP, which ubiquitinates p53 and thereby targets it for degradation^20^,^21^,^48^,^49^. In addition, HPV E6 proteins from high-risk strains target PDZ-domain containing proteins for proteosomal degradation^50^, which is believed to contribute to the malignant transformation of infected cells^3^. Many viral proteins, like HPV E6, have multiple cellular targets. Linking (parts of) such natural targets may be a fertile general strategy to obtain high-affinity binders, as exemplified here for the HPV E6 protein.

## Methods

### Design of constructs

Lipo-E6_18_-C was constructed by PCR-amplification of DNA corresponding to HPV18 E6 C-terminal domain (residues 82-158, UNiProtKB: P06463), from a plasmid encoding the full-length sequence, and insertion of the fragment into a modified pRSET vector (Invitrogen). The expressed construct was made up of an N-terminal His-tagged *Bacillus stearothermophilus* lipoyl protein domain^39^ followed by a thrombin cleavage site (LVPRGS) and finally the C-terminal domain of HPV18 E6. The pseudo wild type SAP97 PDZ2 (residues 311-407) was the same as used in previous experiments^29^,^31^,^32^. The nine different PDZ2 mutants obtained after the phage display selection, designated PDZϕ1-9, were subcloned into the same vector as described for Lipo-E6_18_-C. PDZbodies were constructed by using the plasmid encoding PDZϕ9 as a template. DNA corresponding to the amino acid sequence KLMAAAELTLQELLGEER followed by a Gly-Ser linker ending with Gly-Thr was inserted in between the thrombin cleavage site and Val313 at the N-terminus of PDZϕ9 (numbering of the PDZ domain is according to that of the pseudo wild type SAP97 PDZ2 described above). This inserted sequence includes the LxxLL-motif-containing helix from E6AP used in the recent crystal structure of HPV16 E6^42^. The length of the Gly-Ser linker was 3, 10, 15 and 20 residues, respectively, in four different constructs. This resulted in total linker lengths between the E6AP helix and the first strand of the PDZ domain of 8, 15, 20 and 25 residues, respectively. The PDZbodies were named according to their respective total linker length, i.e. PDZbody20 has a total linker length of 20 residues.

### Phage display-Library construction and selection

Based on the sequence of our pseudo wild type SAP97 PDZ2 construct, a library of the PDZ gene, randomized in the codons for five selected residue positions (His384, Glu385, Val388, Leu391, Lys392, see Fig. 1) and designed to encode all amino acid residues except Cys (to avoid disulfide bridges), was purchased from AbD Serotec as a pool of linear DNA fragments with *Xho*I and *Spe*I restriction sites in the 5′ and 3′ end, respectively. After PCR-amplification of the library and subsequent digestion with the respective restriction enzymes, the DNA was ligated into the pComb3 phagemid pC3scCro8^38^ with a background (undigested pC3scCro8) of <5%. The ligation mixture was transformed into *E. coli* XL1-Blue cells by electroporation and the final library of M13 phages displaying PDZ variants was proliferated, harvested and resuspended essentially as described earlier^38^. The library size was estimated as the number of ampicillin-resistant transformants. Harvested phages were used immediately or stored at 4°C. The phage display affinity selection is described in detail in Supporting Information.

### Expression and purification

Pseudo wild type SAP97 PDZ2, Lipo-E6_18_-C, PDZϕ1-9 and the four PDZbodies 8, 15, 20 and 25, were expressed in *E. coli* BL21(DE3) pLysS (Invitrogen). Transformed cells were grown at 37°C to an optical density = 0.8-1 and protein over-expression was induced with 1 mM isopropyl β-D-thiogalactopyranoside, after which the bacteria were grown overnight at 18°C. Harvested cells were resuspended in Binding Buffer (BB) [50 mM Tris-HCl, pH 8.5, 500 mM NaCl] and lysed by sonication. The samples were then centrifuged for 1 h at 4°C and 35 000 *g*, and the supernatant was filtered through a 0.2 µM filter before loading on a nickel-Sepharose fast flow (GE Healthcare) column. After washing with BB containing 20 mM Imidazole, the His-lipoyl-tagged proteins were eluted with BB containing 250 mM Imidazole. The proteins were then digested overnight with thrombin (ca. 10 units per 15 mg protein) at room temperature before dialysis to Q column buffer (QB) [50 mM Tris-HCl, pH 7.5] followed by loading on a Source 30Q (GE Healthcare) column. The pure proteins were found in the flow through. Their purity was checked on SDS-PAGE stained with Coomassie Brilliant Blue, and their identity confirmed with matrix-assisted laser desorption/ionization time of flight mass spectrometry. For pseudo wild type SAP97 PDZ2 the purification included an intermediate step in which the digested and dialysed sample was supplemented with imidazole to a final concentration of 20 mM and reloaded on the nickel column, after which the flow through was loaded on the Q column. For Lipo-E6_18_-C, purification was done as above with the differences that (*i)* all buffers contained 2 mM β-mercaptoethanol, and (*ii)* after elution from the nickel column thrombin digestion was omitted and (*iii)* the sample was eluted from the nickel column in buffer without NaCl and thereafter diluted three times in QB before loading on the Q column. The pure protein was eluted with a 300 ml gradient of 0-150 mM NaCl in QB buffer. For preparation of samples for measurements with ITC the pure protein sample was supplemented with NaCl to a final concentration of 500 mM and the residual thrombin was removed by passing it through a Benzamidine column (GE Healthcare) equilibrated with QB containing 500 mM NaCl. Full-length HPV16 E6 was expressed and purified as described^42^,^46^.

### Stopped-flow experiments

All of the stopped-flow experiments conducted in order to measure the rate constants for the Lipo-E6_18_-C/PDZ interaction, were performed on an SX-20MV stopped-flow spectrometer (Applied Photophysics Leatherhead, UK) at 10°C in 50 mM potassium phosphate buffer, pH 7.5, 240 µM β-mercaptoethanol. Fluorescence was monitored using the change in emission of the tryptophan Trp342 located in our PDZ variants (excitation at 280 nm; emission at 330 ± 30 nm). The rate constants of association, *k*_on_, and dissociation, *k*_off_, were determined by performing two different sets of experiments. For determination of *k*_on_ we mixed PDZ (varied between 6–20 µM in different experiments) with Lipo-E6_18_-C (2 µM) and fitted the resulting trace of increase in tryptophan emission upon binding to a single exponential equation to obtain the observed rate constant *k*_obs_ at each PDZ concentration. The *k*_obs_ values were plotted *versus* the concentration of PDZ variant and fitted to the general equation for a reversible bimolecular interaction^51^,^52^ from which *k*_on_ was derived. The *k*_off_ was measured with a displacement experiment in which a pre-formed complex of PDZ variant (0.75 µM) and Lipo-E6_18_-C (0.75 µM) was mixed with high concentrations of a dansylated YKQTSV-peptide (20, 150 and 300 µM), which competes with Lipo-E6_18_-C for binding to the PDZ. The observed decrease in tryptophan emission was fitted to a single exponential equation to obtain *k*_obs_. The *k*_obs_ value at high peptide concentration is equal to the overall *k*_off_ value, as described previously^29^.

### ITC experiments

Isothermal titration calorimetry experiments were performed on an ITC200 (Microcal) at 25°C in 20 mM sodium phosphate buffer, pH 6.8, 200 mM NaCl, 1 mM TCEP. Full-length HPV16 E6 protein was titrated into solutions of pseudo wild type SAP97 PDZ2, PDZϕ9, PDZbody8, PDZbody15, PDZbody20 and PDZbody25. The experiments were designed so that the *C* values were within 1–1000 (*C* value = *N* × [Protein]/*K_d_*, where *N* is the stoichiometry of the interaction, [Protein] is the concentration of protein in the cell and *K_d_* is the equilibrium dissociation constant).

We corrected for heat of dilution using the small average effect of the three last injection points. A control experiment in which HPV16 E6 was titrated into buffer also showed small and similar values as the absolute of that average^53^. Origin 7.0 (Microcal) was used to determine the thermodynamic parameters of the HPV16 E6/PDZ interaction using nonlinear least square fitting assuming a single-site model.

### Co-Immunoprecipitation

Plates (10 cm) were seeded with 3×10^6^ HeLa cells in 10 ml DMEM, 10% Foetal Calf Serum, 1% PeSt (penicillin and streptomycin). After 24 h the cells were transfected with empty p3XFLAG_CMV vector (encoding a triple FLAG-tag) as a control, or the same vector expressing the pPDZbody20 with a triple FLAG-tag in the C-terminus. using Turbofect (Thermo Scientific), according to recommendations. Briefly, 10 μg plasmid was mixed with 1 ml RPMI media. After vortexing, 20 μl Turbofect was added, the mix was further vortexed, incubated for 20 min and then added dropwise to the cells. After another 24 h the transfections were harvested using 750 μl lysis buffer/plate, according to protocol (SIGMA, ANTI-FLAG M2 Affinity Gel, #A2220). Protein levels in the lysates were determined using the BCA protein assay (Pierce Biotechnology) and a total amount of 1.5 mg was routinely obtained. Lysate corresponding to a total of 1 mg of protein was used per sample in subsequent co-immunoprecipitation experiments. Co-immunoprecipitation was performed according to the manufacturers recommendations (SIGMA, #A2220). Briefly, FLAG-tag antibody (M2)-beads were washed in 1×TBS (50 mM Tris-HCl, with 150 mM NaCl, pH 7.4) and then incubated with 3% (w/v) bovine serum albumin in 1×TBS for 1 h at 4°C. After washing the beads with 1×TBS they were divided between the samples and incubated for 24 h at 4°C. After washing in 1×TBS, bound proteins were eluted by boiling the beads with 15 μl protein loading dye for 5 min. The samples were centrifuged to remove any precipitate and the proteins in the supernatant were loaded and separated on AnyKD page gels (Mini-protean TGX, 4–20%, BIO-RAD).

### Western blotting

The samples were transferred onto PVDF membranes by wet blotting at 180 mA for 1 h. The membranes were incubated with Odyssey Blocking Buffer (PBS based) (LI-COR Biosciences) for 1 h at 22°C. After a brief rinse in 1×PBS the membranes were incubated overnight at 4°C with primary antibodies, HPV18-E6 (1:100 dilution, sc-365089, Santa Cruz), FLAG-tag (1:2500 dilution, M2, SIGMA) and anti-actin (1:2500 dilution, sc-1616, Santa Cruz) diluted in Blocking Buffer. Secondary antibodies used were anti-mouse-IRDye800 (LI-COR Biosciences) and anti-goat-IRDye800 (LI-COR Biosciences) diluted in Blocking Buffer. After incubation with primary and secondary antibodies the membranes were washed for 3×20 min with 1×PBS. The membranes were analysed in an Odyssey Imager (LI-COR Biosciences).

## Author Contributions

A.K. and P.J. conceived the project. A.K., J.R., D.Ö., T.M., C.C., M.W., G.T., M.N. and P.J. planned the experiments. A.K., J.R., D.Ö., T.M., Å.E., and M.F. performed the experiments. A.K., J.R., D.Ö., T.M., G.T., M.N. and P.J. analysed the data. A.K., T.M., D.Ö. and P.J. wrote the paper. All authors reviewed the final manuscript.

## Supplementary Material

Supplementary Information Supplementary information

## Figures and Tables

**Table 1:** PDZ variants selected by phage display towards the C-terminal domain of HPV18 E6 together with their binding rate constants and affinities for Lipo-E6_18_-C

PDZ variant	Mutations	*k*_off_ (s^−1^)	*k*_on_ (µM^−1^s^−1^)	*K*_d_ (μM)
Pseudo wild type	(9)*1*	3.3 ± 0.01*2*	6.2 ± 0.05*2*	0.54 ± 0.01*2*
PDZϕ1	E385R (3)	7.5 ± 0.09	8.2 ± 0.1	0.92 ± 0.02
PDZϕ2	E385H, L391F, K392Q (1)	1.5 ± 0.01	5.9 ± 0.08	0.26 ± 0.01
PDZϕ3	L391F, K392A (1)	0.75 ± 0.003	4.6 ± 0.03	0.16 ± 0.01
PDZϕ4	E385H, K392R (4)	5.1 ± 0.05	8.5 ± 0.1	0.60 ± 0.02
PDZϕ5	E385H, K392T (1)	3.8 ± 0.03	4.9 ± 0.05	0.78 ± 0.01
PDZϕ6	E385K, K392Q (1)	10.2 ± 0.3	4.0 ± 0.08	2.5 ± 0.03
PDZϕ7	E385K, K392R (1)	5.5 ± 0.06	8.9 ± 0.13	0.61 ± 0.02
PDZϕ8	E385T (2)	3.8 ± 0.04	5.8 ± 0.06	0.66 ± 0.01
PDZϕ9	L391F, K392M (1)	0.55 ± 0.002	5.9 ± 0.04	0.093 ± 0.008

^1^The number in parenthesis equals the number of a particular variant among the 24 sequenced after the phage display selection.

^2^Value±standard error (*k*_off_ and *k*_on_); the error for *K*_d_ is the propagated standard error (*K*_d _ = *k*_off_/*k*_on_).

**Table 2:** Parameters from ITC measurements of binding between PDZ variants and full-length HPV16 E6

PDZ variant	*ΔH* (kcal mol^−1^)	*n*	*K*_d_ (μM)	*ΔΔG*^variant-pWT^ (kcal mol^−1^)
Pseudo wild type (pWT)	−4.6 ± 0.5*1*	0.75 ± 0.05*1*	6.3 ± 1.3*1*	-
PDZϕ9	−9.9 ± 0.3	0.75 ± 0.02	0.91 ± 0.12	−1.2 ± 0.15*1*
PDZbody8	−19.1 ± 0.1	0.76 ± 0.004	0.20 ± 0.01	−2.1 ± 0.13
PDZbody15	−16.5 ± 0.1	0.93 ± 0.004	0.16 ± 0.01	−2.2 ± 0.13
PDZbody20	−17.0 ± 0.2	0.94 ± 0.007	0.065 ± 0.012	−2.7 ± 0.16
PDZbody25	−19.8 ± 0.11	0.92 ± 0.003	0.079 ± 0.006	−2.6 ± 0.13

^1^Value±standard error (*ΔH, n* and *K*_d_); the error for *ΔΔG*^variant-pWT ^is the propagated standard error (*ΔΔG*^variant-pWT^ *= RT ln(K*_d_^variant^/*K*_d_^pWT^*)*).
