# Supplementary material for: Design of a PDZbody, a bivalent binder of the E6 protein from human papillomavirus
Source: Sci Rep. 2015 Mar 23;5:9382. doi: 10.1038/srep09382 (PMC4369733; doi:10.1038/srep09382)
Supplement: Supplementary Information [file srep09382-s1.pdf]

SUPPORTING INFORMATION

Design of a PDZbody, a bivalent binder of the E6 protein from human papillomavirus

O. Andreas Karlsson[‡], Juan Ramirez[§], Daniel Öberg[‡], Tony Malmqvist[‡], Åke Engström[‡], Maria Friberg[‡], Celestine N. Chi[‡], Mikael Widersten[¶], Gilles Travé[§], Mikael T. I. Nilsson[¶], and Per Jemth[‡*]

[‡]Department of Medical Biochemistry and Microbiology, Uppsala University, BMC Box 582, SE-75123 Uppsala, Sweden.

[§]Biotechnologie et Signalisation Cellulaire UMR 7242, Ecole Supérieure de Biotechnologie de Strasbourg, Boulevard Sébastien Brant, BP 10413, F-67412 Illkirch, France.

[¶]Department of Chemistry-BMC, Uppsala University, Box 576, SE-751 23 Uppsala, Sweden.

[*]Corresponding author: Per Jemth, E-mail: Per.Jemth@imbim.uu.se

CONTENTS

Supplementary text. Detailed description of the phage display affinity selection to support the main text.

Phage display-Affinity selection

In order to avoid enrichment of the <5% scCro8-displaying phages during selection, the library was preincubated for 30 minutes at room temperature with 1.7 μM of the scCro8 DNA ligand ORC[1] prior to a 1 h incubation at 4 °C with 2 μM Lipo-E6$_{18}$-C in a final volume of 100 μl in binding buffer (BB) [10 mM Tris-HCl pH 7.4, 100 mM KCl, 2 mM β-mercaptoethanol], resulting in a final concentration of 1 μM ORC. The mixture was thereafter transferred to paramagnetic precharged nickel beads (Promega) washed with BB containing 0.5% (w/v) bovine serum albumin (BSA) and capable of binding 5-10 times the amount of His-tagged Lipo-E6$_{18}$-C present. After 2-5 minutes of incubation at room temperature the beads were captured and washed for approximately 15 seconds with 500 μl of ice-cold wash buffer (WB) [BB containing 0.3 % (w/v) BSA and 1 μM ORC] before transfer to a BSA-blocked tube in which the wash was repeated twice. The beads and bound phages that passed the selection were resuspended in 2×50 μl 2TY [1.6% (w/v) Peptone, 1% (w/v) yeast extract, 0.5% (w/v) NaCl] and transferred to a sterile plastic tube. Five ml log phase culture of *E. coli* XL1-Blue grown in 2TY (containing 10 μg/ml of tetracycline) were added to the surviving phages, which were allowed to infect the bacteria for a total of 15 minutes. The phages were then proliferated as described above while the number of surviving phages (ϕ_{out}) was estimated by titering the infected and thereby ampicillin resistant bacteria. The enrichment during selection was monitored as ϕ_{out}/ϕ_{in}, *i.e.*, the number of surviving phages divided by the number of phages entering the selection (ϕ_{in}). The selection procedure was repeated for a total of five rounds, with the following modifications: For rounds 2-5 the amounts of both beads and Lipo-E6$_{18}$-C were lowered five times as compared to the first round. The final wash in the blocked tube was performed six and ten times for round two and three respectively. For rounds four

and five BB was changed to 1×PBS with 2 mM β-mercaptoethanol and (*i*) a pre-selection towards biotinylated ORC (b-ORC)[1] was performed in order to further avoid enrichment of scCro8, as well as (*ii*) a finishing step with the presence of a HPV18 E6 n C-terminal peptide (LQRRRETQV) to preferentially enrich phages displaying a PDZ variant with low rate of dissociation from Lipo-$E6_{18}$-C. For the pre-selection, proliferated survivors were incubated with 1.7 μM b-ORC for 30 minutes at room temperature and the unwanted bound phages were captured with 0.1 mg streptavidin-coated paramagnetic beads (Promega). The remaining unbound phages then entered the regular selection. After transfer to the BSA-blocked tube the finishing step was performed. The beads were washed with 3×1 ml of ice-cold WB, resuspended in 100 μl BB containing 40 μM LQRRRETQV peptide, and incubated at room temperature during 4 minutes. After another wash with 3×1 ml of ice-cold WB, the phages that passed the selection were treated as described above.

(1) Nilsson, M. T., Mossing, M. C., and Widersten, M. (2000) Functional expression and affinity selection of single-chain cro by phage display: isolation of novel DNA-binding proteins. *Protein Eng. 13*, 519–526.
